# Endogenous retroviruses and multiple sclerosis–new pieces to the puzzle

**DOI:** 10.1186/1471-2377-13-111

**Published:** 2013-08-28

**Authors:** Kari K Nissen, Magdalena J Laska, Bettina Hansen, Thorkild Terkelsen, Palle Villesen, Shervin Bahrami, Thor Petersen, Finn S Pedersen, Bjørn A Nexø

**Affiliations:** 1 Department of Biomedicine, Aarhus University, Wilhelm Meyers Allé 4, DK-8000, Aarhus, C, Denmark; 2 Bioinformatics Research Centre, Aarhus University, C.F.Møllers Allé 8, DK-8000, Aarhus, C, Denmark; 3 SKAUvaccines, Incuba Science Park, Åbogade 15, DK-8200, Aarhus, N, Denmark; 4 Department of Neurology, Aarhus University Hospital, Nørrebrogade 44, DK-8000, Aarhus, C, Denmark; 5 Department of Molecular Biology and Genetics, Aarhus University, C.F.Møllers Allé 3, DK-8000, Aarhus, C, Denmark

**Keywords:** Multiple sclerosis, Endogenous retroviruses, HERV-Fc1, TRIM, BST2, Genetic association

## Abstract

The possibility that retroviruses play a role in multiple sclerosis (MS) has long been considered; accumulating findings suggest this to be most likely in the form of human endogenous retroviruses (HERVs). A genetic test series of fifty endogenous retroviral loci for association with MS in Danes showed SNP markers near a specific endogenous retroviral locus, HERV-Fc1 located on the X-chromosome, to be positive. Bout Onset MS was associated with the HERV-Fc1 locus, while a rarer form, Primary Progressive MS, was not. Moreover, HERV-Fc1 *Gag* RNA in plasma was increased 4-fold in patients with recent history of attacks, relative to patients in a stable state and to healthy controls.

Finally, genetic variations in restriction genes for retroviruses influence the risk of MS, providing further support for a role of retroviral elements in disease.

We speculate that endogenous retroviruses may activate the innate immune system in a variety of ways, involving the host proteins, TRIMs, TLRs, TREXs and STING. Observations in HIV-positive patients suggest that antiretroviral drugs can curb MS. Thus, these new findings regarding the etiology and pathogenesis of MS, suggest alternative ways to challenge autoimmune diseases.

## Review

Multiple sclerosis (MS) is a chronic inflammatory, demyelinating disease of the central nervous system, probably caused by interaction of multiple genes and environmental factors. It is the most common neurological disease causing debilitation in young people in Scandinavia. Disease onset usually occurs in young adults, and it is more common in women [1]. It has a prevalence that ranges between 2 and 150 per 100,000 [2]. The ultimate pathogenic effector appears to be the immune system [1]. In most people, MS is initially characterized by sudden bouts of disease, called attacks, alternating with periods of remission. Gradually, the disease may become progressive. There is no known cure for multiple sclerosis and treatments attempt to return function after an attack, prevent new attacks, and prevent disability [1].

MS has a clear genetic component as evidenced by family studies [3,4] and studies of twins [5]. The human leukocyte antigen (HLA) immune gene-cluster is long established as a risk factor [6], and recent genome wide association studies (GWAS) have added numerous susceptibility loci, mainly related to immune regulatory genes, to the list [7]. However, each of these contributes modest effects to the combined risk of MS development, and still leaves genetic risk factors to be identified [8]. Many environmental factors, from sunlight to smoking, have been suggested [9]. Some epidemiological findings could suggest the contribution of infectious agents [10], and especially infection with Epstein-Barr virus seems to predispose to MS later in life [11]. MS is thus assumed to develop in genetically susceptible individuals after exposure to certain environmental risk factors.

The possibility that retroviruses play a role in the pathogenesis of MS has long been considered [12]. In several animal models (sheep, mice, and monkeys) demyelinating diseases are well known, and can be caused by horizontally transmitted retroviruses [13-15]. A number of retroviruses have been considered since they have links with similar neurodegenerative disorders, such as HTLV-1, which causes tropical spastic paraparesis in humans [16], and the Visna-Maedi virus, which causes an MS-like disease in sheep [17]. This led to intense search for a retrovirus involved in MS and in 1989 retroviral particles and reverse transcriptase activity was detected in cells from MS patients [18]. Further studies of this MS-associated retrovirus (MSRV) finally established it to be of endogenous origin, forming a new family of HERV-W [19]. Human endogenous retroviruses (HERVs) are remnants of prehistoric exogenous retroviruses, integrated in the human genome during primate evolution through germ line infection.

At present, there is no direct evidence for the involvement of an exogenous retrovirus in MS, but endogenous retroviruses may well be one or more of the genetic factors in the pathogenesis. As will become apparent in the following, we do not see the endogenous retroviruses as alternatives to the immune-mediated damage, but as triggers of it.

Over time, a large number of studies have reported the detection of various HERV-molecules (mainly from HERV-W and HERV-H) and antibodies towards such epitopes more frequently in MS patients, as was recently reviewed by Antony *et al*. [20]. An intriguing new development has been the report of increased numbers of HERV-W DNA copies in genomic DNA from PBMCs from secondary progressive MS patients, suggesting that HERV-W amplifies in these patients [21].

A potential problem in the implication of HERV-W/MSRV and HERV-H sequences in MS from the studies described above is the inability to distinguish causation and passenger status through expression experiments. Does the virus contribute to disease or does disease activate the virus? In spite of the importance of these findings, this is a problem that cannot be solved within the frame of expression studies of ubiquitous genomic elements. To get around this obstacle, we initially chose to study the genetics of endogenous retroviral loci, followed by physiological experiments only when the identity of a genetically associated locus was established. As far as is known, the disease cannot alter specific polymorphisms in polyclonal DNA, which is used in these studies. The direction of causality is thus relatively certain to be from DNA to disease; not the reverse.

By means of an approach based on genetic epidemiology, single nucleotide polymorphisms (SNPs) in close proximity of fifty endogenous retroviral loci were tested for statistical association with MS. The fifty loci were chosen because their sequences indicate that between zero and two mutations would enable encoding at least one full length viral protein. Markers near a specific endogenous retroviral locus, HERV-Fc1, located on the human X-chromosome, were found to be associated with disease in a Danish cohort of 350 MS cases and 500 controls [22]. HERV-Fc1 had not previously been related to MS. The association was repeated in one Danish, and in one Norwegian cohort [22,23]. A fourth (Danish) cohort was negative for disease association [22].

Subtypes of MS have also been studied for association with HERV-Fc1. Bout Onset MS, the common forms of MS encompassing Relapsing/Remitting MS and Secondary Progressive MS, was associated with the locus, while another form of MS, Primary Progressive MS, seemed not to be [23]. Interestingly, an independent study has indicated that Primary Progressive MS may be associated with another endogenous retroviral locus at chromosome 7, HERV-16 (SNP rs996343) [24]. In a series of expression studies [25], it was shown that the level of HERV-H/F GAG protein (determined by antibodies targeting but not specific to HERV-Fc1) is increased in PBMCs from MS patients relative to healthy controls. It was also found that the level of protein was elevated in the circulating T-cell compartment in MS patients with a recent history of attacks, relative to patients in a stable state and to healthy controls. Finally, in agreement with this it was found that expression of HERV-Fc1 RNA in plasma was increased 4-fold in patients with a recent history of attacks, relative to patients in a stable state and to healthy controls. Combined with the genetic studies this suggests an active role of HERV-Fc1 in the pathogenesis of MS. A search for extra germ line copies of HERV-Fc1 in MS was unfruitful [26].

Treatment with 5-aza-dC (5-azadeoxycytidine) resulted in demethylation of HERV-Fc1 5′LTR and significantly increased levels (up to 50 000 fold) of HERV-Fc1 mRNA expression in cells previously not expressing HERV-Fc1, or with a very low basic expression level [27,28]. This confirms that retroelements are generally epigenetically silenced in somatic cells [29]. Although we have no data as of yet, one could imagine that other physiological mechanisms might also activate expression of HERV products and could contribute to disease.

Another line of inquiry supports the involvement of retrovirus in MS. Genes known to restrict the replication of viruses, namely *TRIM5*, *TRIM22* and *BST2*, have been shown to influence the risk of MS [22], and Nexø et. al manuscript submitted]. The so-called *APOBEC3* genes showed a similar, but statistically insignificant, tendency, while the *TREX1* gene seemed inert. These retroviral restriction factors, protecting the host from exogenous retroviral infection, may also recognize products of endogenous proviruses [30-33].

Current models of MS pathogenesis implicate the immune system as the ultimate effector, but combine it with neurodegeneration [34]. How this might be related to the endogenous retroviruses is unknown, but several possibilities can be suggested. HERVs in general are defective. However, since some are able to produce proteins they may be able to start an infectious-like process either through complementation or recombination. Thus, it might be the entire endogenous retroviral repertoire that determines infectivity and not the individual locus. If activated, the endogenous viruses could trigger the adaptive immune system or, equally likely, the innate immune system.

The human genome contains several sets of genes encoding dozens of proteins constituting anti-retroviral defense mechanisms. One such set includes the TRIM proteins. TRIM5 protein activation is mediated by the incoming viral capsid. Recently, Jeremy Luban’s group reported that TRIM5-binding of the capsid activates signal transduction, stimulating the innate immune system [35]. As described above, markers in and around the *TRIM5* gene are associated with the risk of MS [22] and Nexø *et al*. manuscript submitted]. Thus, it would be interesting to investigate the possibility that HERVs with potential Gag expression, e.g. HERV-Fc1, via TRIM could stimulate the innate immune system.

Alternative mechanisms for the activation of the innate immune system may exist. Un-integrated DNA in the cytosol is known to trigger innate immune signaling [36]. RNA components of internalized viral sequences activate the innate immune system by stimulating Toll-like receptors in the endosomes [37]. The triggering of TLRs by HERVs in MS has previously been proposed [38]. Moreover, triggering of the innate cellular antiviral system by membrane-fusion of nucleic acid devoid virus-like-particles and cell, may act via the adaptor STING [39]. Finally, an entirely different mechanism was described in a recent paper; Dysregulation of the heterochromatin factor HP1α binding sites was shown to occur in MS. HP1α was again shown to influence both expression of HERVs and immune factors [40].

Thus, the endogenous viruses are mobile subcellular structures that span the divide between self and foreign. We specifically suggest that HERVs, though now a part of self, to some extent have retained the ability to trigger innate immune sensors of foreign patterns. This in turn could lead to stimulation of an adaptive autoimmune response. A combination of the above mechanisms could conceivably lead to a response, initially elicited by the endogenous viruses, but ultimately reacting to a broad range of cellular components.

Noticeably, none of these models require actual productive infection cycles; a steady supply of virus-like particles performing the initial parts of an infection would be enough. Therefore, our induction experiments, which show that high levels of viral mRNA expression can be achieved from the endogenous loci when cells are exposed to drugs, take on a special significance. However, we sorely miss the identification of normo-physiological mechanisms other than actual replication that can enhance endogenous virus expression to an extent leading to activation of the immune system. Late infection with Epstein Barr Virus (EBV) is associated with MS, and *in vitro* EBV infection of blood leucocytes and astrocytes leads to activation of HERVs [41]. Maybe, late EBV infection *in vivo* triggers large scale HERV activation.

Future investigations must substantiate the involvement of HERVs in MS by genetic experiments as well as expression studies. Studies of host factors should serve the same purpose. The activation of the innate immune system by retroviruses and the role of *TRIMs*, *TLRs*, *TREXs*, *STING* and *HP1α* should be elucidated in both genetic and expression studies. The potential interaction between different HERVs is also of considerable interest.

Importantly, recent data suggest that MS is influenced by highly active anti-retroviral treatment (HAART) [42,43]. MS is uncommon among HIV-positive HAART-treated persons (incidence rate ratio = 0.3: 95% confidence interval = 0.04-2.2). Although the group-size is small, and the results are not statistically significant, the effect is fairly strong and in accordance with the expected trend suggesting that antiretroviral medicines can curb MS. If these findings are substantiated, it becomes imperative to investigate in a clinical trial, if HAART can be effective against MS and possibly other autoimmune diseases. At the moment, a clinical trial for the antiviral drug Raltegravir is in its initial phase, to test if this integrase inhibitor can suppress HERV activity and ameliorate MS progression [44]. A more targeted approach is also on trial, as a monoclonal antibody towards MSRV Env (GNbAC1) is being tested as a remedy for MS [45]. It will be most interesting to follow these innovative treatment strategies.

## Conclusion

Solid evidence points to an involvement of the endogenous retrovirus HERV-Fc1 in the initiation of MS in Scandinavians. Time will show if the more speculative aspects of this review are borne out.

## Competing interests

The authors declare that they have no competing interests.

## Authors’ contributions

KKN, MJL, BAN: Ideas, drafting. All other authors: Contributory ideas, discussions, review of the manuscript, literature search. All authors read and approved the final manuscript.

## Pre-publication history

The pre-publication history for this paper can be accessed here:

http://www.biomedcentral.com/1471-2377/13/111/prepub
